# Experimental analyzing the effect of n-heptane concentration and angular frequency on the viscoelastic behavior of crude oil containing asphaltene

**DOI:** 10.1038/s41598-022-07912-y

**Published:** 2022-03-10

**Authors:** Mohammadjavad Fazeli, Mehdi Escrochi, Zohreh Sadat Hosseini, Behzad Vaferi

**Affiliations:** 1 Department of Chemical Engineering, Shiraz Branch, Islamic Azad University, Shiraz, Iran; 2 Department of Petroleum Engineering, School of Chemical and Petroleum Engineering, Shiraz University, Shiraz, Iran; 3 Department of Chemical Engineering, School of Chemical and Petroleum Engineering, Shiraz University, Shiraz, Iran

**Keywords:** Chemistry, Engineering, Materials science

## Abstract

Asphaltene often produces problems in upstream and downstream sections of crude oil transportation and processing equipment. These issues are directly related to the asphaltene precipitation in transportation pipelines, separation columns, heat exchangers, and storage tanks. This research investigates the impact of angular frequency and n-heptane concentration on asphaltene precipitation and rheological behavior of two oil samples from the Mansouri oil field in Iran, i.e., 23 and 71. The viscosity tests revealed that these oil samples and their mixtures with n-heptane exhibit Newtonian behavior. Moreover, increasing the n-heptane concentration increases the asphaltene precipitation and dramatically decreases crude oil viscosity. The frequency tests revealed that the presence of n-heptane has an unfavorable effect on crude oil’s viscoelastic behavior. Therefore, it is necessary to find the optimum range of angular frequency and n-heptane concentration to minimize the asphaltene content of crude oil and provide them with appropriate viscoelastic behavior. Increasing the angular frequency continuously increases all oil samples’ loss modulus and strengthens their liquid-like manner. The experimental results confirmed that the angular frequency higher than 33.6 rad/s and 75% volume concentration of n-heptane is the best condition for the oil sample of 23. On the other hand, the angular frequency higher than 23.4 rad/s and 75% volume concentration of n-heptane is the best condition for the oil sample of 71. In these conditions, the oil samples of 23 and 71 not only have appropriate viscoelastic behavior, but they also experience 97.2% and 96.3% reductions in their viscosity, respectively.

## Introduction

These days, crude oil-derived fuels have undeniable roles in providing domestic and industrial activities with their energy demand^1^. Increasing demand for fossil fuels leads to conducting so many studies on different aspects of the petroleum reservoirs, such as drilling^2^, fluid extraction^3,4^, pressure maintenance^5^ and analysis^6–8^, formation characterization^9^, enhanced oil recovery^10–12^. The presence of water emulsion is one of the main problems for crude oil transportation and its processing^13,14^. This water emulsion creates undesirable operation problems, including pipeline corrosion and blockage through hydrate formation, increasing pressure drop, increasing transportation cost, and deactivating catalysts^15,16^. The stability of water emulsion is due to the elastic interfacial film resulting from the asphaltene content of crude oil^14^. Asphaltene is the heaviest fraction of crude oil, consisting of the aromatic and aliphatic part^17^. These heavy materials also have the heteroatom part, forming hydrogen bonds with the water molecules and stabilizing asphaltenes at the oil–water interface^18^. The cyclic structure of this heavy fraction results in its solubility in aromatic compounds like toluene and benzene and their insolubility in n-alkanes such as n-heptane^19^. On the other hand, asphaltene precipitation often leads to severe problems in crude oil’s transportation and processing stages^20,21^. Asphaltene separation from crude oil and its degradation is one of the practical methods to overcome the problems raised by asphaltene^22–24^. Normal alkanes are among the most well-known materials used as asphaltene precipitation additives^25^.

A comprehensive study of the rheological behavior of crude oil containing asphaltene can provide helpful information to decrease/postpone asphaltene precipitation. Fan et al. investigated the effect of oil aromaticity, time, asphaltene concentration, and nonionic surface-active additives on water–oil emulsion stability and its interfacial properties^26^. Results justified that water–oil emulsion’s stability decreased by increasing oil aromaticity. Moreover, nonionic surface-active additives prevent the formation of asphaltene clusters.

Wave-based analyses have recently been suggested for studying systems’ behavior^27–29^. Rane et al. experimentally studied the effect of angular frequencies on elastic modulus of oil contating asphaltene^30^. The results showed that the elastic modulus increases by increasing the frequency first and then becomes constant. Tao et al. investigated the rheological properties of the water–oil emulsions interface and its decomposition^31^. The results showed that loss modulus is directly related to the viscosity and can influence the emulsion’s decomposition. Moreover, dynamic interfacial tension was introduced as the main factor for controlling the emulsions. Garcia-Olvera et al. illustrated the effect of acidic and asphaltenic content on interfacial rheology of crude oil^32^. The interfacial viscoelasticity increases by increasing asphaltene concentration and decreases by reducing the acidic content. Rogel et al. studied asphaltene precipitates in Venezuelan and Canadian heavy crude oils in the presence of benzoyl peroxide^33^. They claimed that benzoyl peroxide is the most effective additive for the considered task. Campen et al. worked on the influence of the toluene-heptane mixture on asphaltene precipitation^34^. They declared that the asphaltene precipitation rate and size increases by increasing the heptane/toluene ratio. Kuang et al. designed a packed bed column to study the effect of temperature, pressure, and precipitating agent on the asphaltene separation^35^. The results showed that the asphaltene formation onset was postponed by increasing the precipitant concentration. Casas et al. studied the rate of asphaltene flocculation in the presence of normal heptane and normal pentane^36^. They stated that the asphaltene precipitation rate increases remarkably by adding 75% n-alkane. Higher concentrations of n-alkane produce a reverse trend in the asphaltene precipitation rate. A summary of previous studies and their main findings have been presented in Table 1.

**Table 1:** A summary of previous studies and findings.

Researchers	Base of study	Main findings
Fan et al.^26^	Water–oil emulsion stability & its interfacial properties	Stability decreased by increasing oil aromaticity & Nonionic surface-active additives prevent the formation of asphaltene clusters
Rane et al.^30^	Elastic modulus at different frequencies	Elastic modulus increases by increasing the frequency at first and then becomes constant
Tao et al.^31^	Rheological properties of the water–oil interface	Loss modulus is directly related to the viscosity and can influence the emulsions decomposition
Garcia-Olvera et al.^32^	Effect of acidic and asphaltenic content on interfacial rheology	Interfacial viscoelasticity increases by increasing asphaltene concentration and decreases by reducing the acidic content
Rogel et al.^33^	Asphaltene precipitates in the presence of benzoyl peroxide	They claimed that benzoyl peroxide is the most effective additive
Campen et al.^34^	Influence of toluene and normal heptane mixture on asphaltene precipitation	Asphaltene precipitation rate and size increase by increasing the heptane/toluene ratio
Kuang et al.^35^	Effect of agent on the asphaltene precipitation	Asphaltene formation onset was postponed by increasing the precipitant concentration
Casas et al.^36^	Effect of n-heptane and n-pentane on asphaltene flocculation	Asphaltene precipitation rate increases remarkably by increasing the n-alkane concentration up to 75%

**Figure 1:**
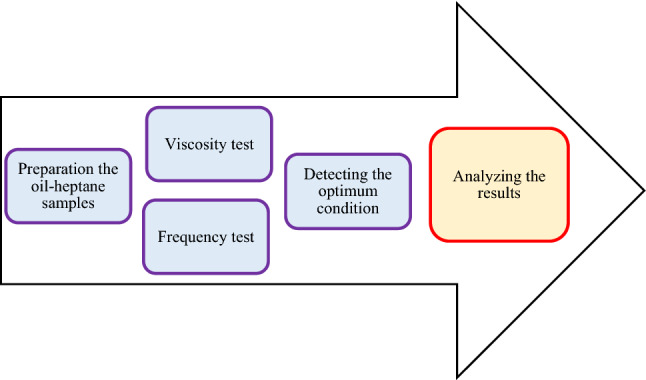
Procedure of the present study.

The current study investigates the effect of n-heptane concentration and angular frequency on the viscoelastic behavior of two oil samples from the Mansouri oil field. Since asphaltene is not soluble in n-heptane, oil mixing with n-alkane helps asphaltene precipitation. This study’s first goal is to find the optimum condition (n-heptane concentration and angular frequency) where these samples show the appropriate viscoelastic behavior. Moreover, we intend to eliminate the asphaltene content of crude oil through precipitation as much as possible. Figure 1 is a schematic that represents the summary of the current study.

## Materials and methods

In the experimental stage of this study, eight oil samples containing different volume concentrations of n-heptane are prepared. Tables 2 and 3 report the specification of these eight oil/n-heptane samples.

**Table 2:** Composition of the prepared samples based on oil type 23.

Oil sample	23–00*	23–25	23–50	23–75
Vol% of oil type 23	100	75	50	25
Vol% of n-heptane	0	25	50	75

*Volume percent of n-heptane in oil type 23.

**Table 3:** Specification of prepared samples based on oil type 71.

Oil samples	71–00*	71–25	71–50	71–75
Vol% of oil type 71	100	75	50	25
Vol% of n-heptane	0	25	50	75

*Volume percent of n-heptane in oil type 71.

The Anton Paar MCR 302 rheometer is utilized to conduct viscosity and frequency tests on the synthesized oil/n-heptane samples. These tests can reveal the asphaltene content and rheological behavior of the samples. More specifically, the viscosity test monitors the effect of shear rate and n-heptane concentration on the oil samples’ viscosity. After that, it is possible to deduce the asphaltene content of the oil/n-heptane samples.

**Figure 2:**
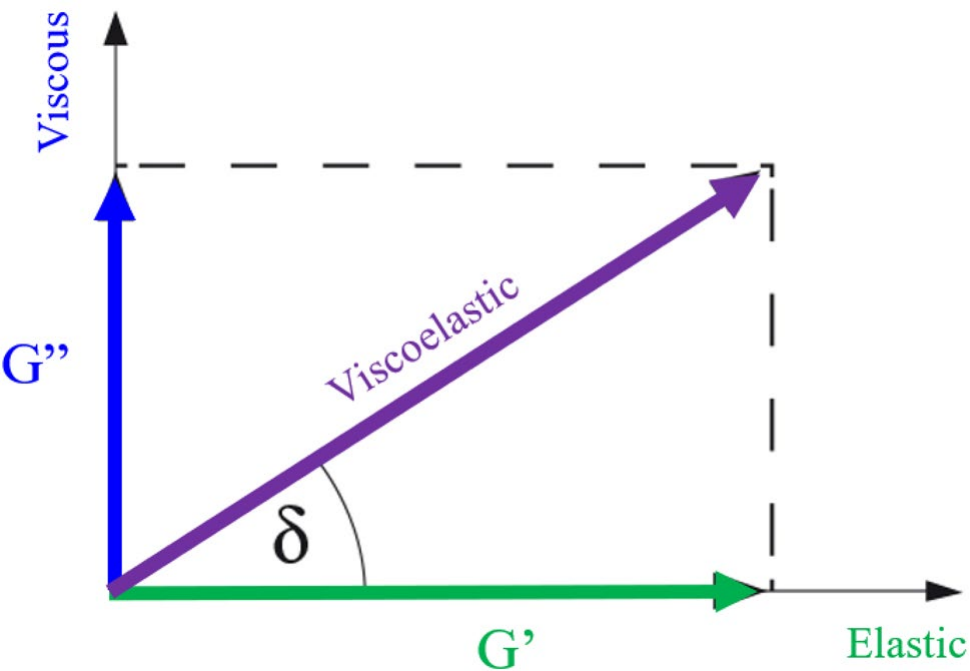
Storage (G′) and loss (G″) modulus.

On the other hand, the frequency test is carried out to observe the viscoelastic behavior of the synthesized oil/n-heptane samples. Indeed, we focused on the storage and loss modulus of the samples to study their viscoelastic behaviors. As Fig. 2 shows, the storage or elastic modulus (G′) represents the elastic portion of the viscoelastic behavior, and it describes the sample’s solid-like behavior. In contrast, the loss or viscous modulus (G″) is associated with viscous and liquid-like behavior.

Furthermore, the viscous modulus to elastic modulus ratio (i.e., loss factor or damping factor) is proposed as a criterion for determining the fluid’s solid or liquid prevailing behavior (Eq. 1). For a fluid with ideal elastic characteristics, the viscous behavior portion is zero, and the loss factor ($\delta$) equals zero. On the other hand, samples with an exclusive viscous property have a loss factor that converges to infinity. In practice, the sample having a loss factor of less than one is considered an ideal elastic fluid with a solid-like behavior. Conversely, the sample with a loss factor of more than 100 is an ideal viscous fluid with a liquid-like behavior.

1 $$\tan \delta = \frac{{{\text{G}}^{\prime } }}{{{\text{G}}^{\prime \prime } }}$$

## Results and discussion

It is better to note that all tests have been conducted at a constant temperature (i.e., ambient temperature). Therefore, the effect of temperature on the viscosity of the samples can be ignored. Indeed, the temperature-dependent nature of the viscosity has no effect on the results.

### Viscosity test

**Figure 3:**
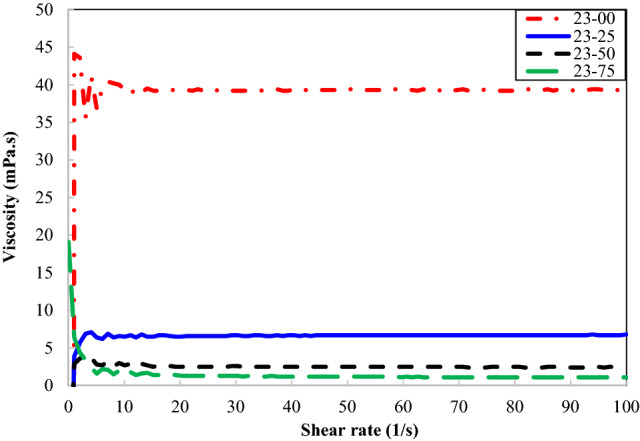
Viscous behavior of different concentrations of n-heptane in oil type 23.

**Figure 4:**
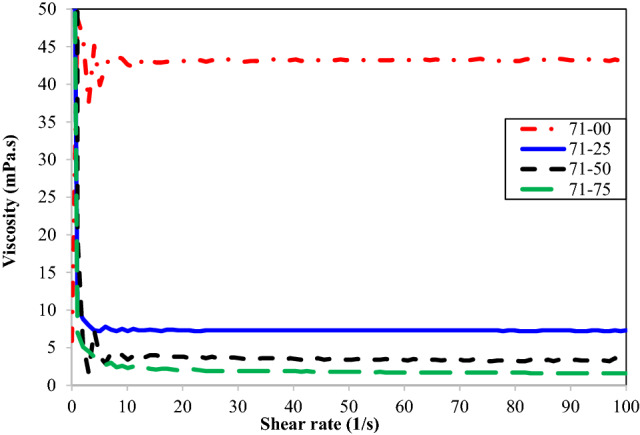
Viscosity of different concentrations of n-heptane in oil type 71.

The viscosity test results conducted on the samples with different concentrations of n-heptane are depicted in Figs. 3 and 4. These figures confirm that all oil samples show the Newtonian behavior, and their viscosities remain almost constant at different shear rates. Furthermore, the viscosity continuously decreases by increasing the n-heptane concentration. Asphaltene coagulation/precipitation due to its insolubility in n-heptane may be introduced as the main reason for this viscosity reduction. Reducing the asphaltene content of the samples leads to a decrease in the residual oil viscosity.

Dynamic viscosity of oil samples containing different concentrations of n-heptane in oil types of 23 and 71 is reported in Tables 4 and 5, respectively. Moreover, the viscosity reduction of the samples due to n-heptane concentration has been reported in these tables. It is not hard to see that the oil samples containing the highest n-heptane concentration experience the highest viscosity reduction. It can be concluded that adding 75% volume concentration of n-heptane to oil types 23 and 71 significantly reduces their viscosity and provide them with a more desirable rheological behavior. This range of n-heptane reduces the viscosity of oil types 23 and 71 by 97.2% and 96.3%, respectively.

**Table 4:** Effect of n-heptane concentration on the viscosity of oil type 23.

Oil samples	23–00	23–25	23–50	23–75
Viscosity (mPa.s)	39.3	7.6	2.5	1.1
Viscosity reduction (%)	–	80.6	93.6	97.2

**Table 5:** Effect of n-heptane concentration on the viscosity of oil type 71.

Oil sample	71–00	71–25	71–50	71–75
Viscosity (mPa.s)	43.2	7.3	3.3	1.6
Viscosity reduction (%)	–	83.1	92.3	96.3

### Frequency test

This section aims to study the effects of n-heptane on the rheological properties of the oil types 23 and 71. The viscoelastic behavior of oil samples of 23 and 71 containing different volume concentrations of n-heptane is monitored using the frequency test.

#### Oil/n-heptane samples

**Figure 5:**
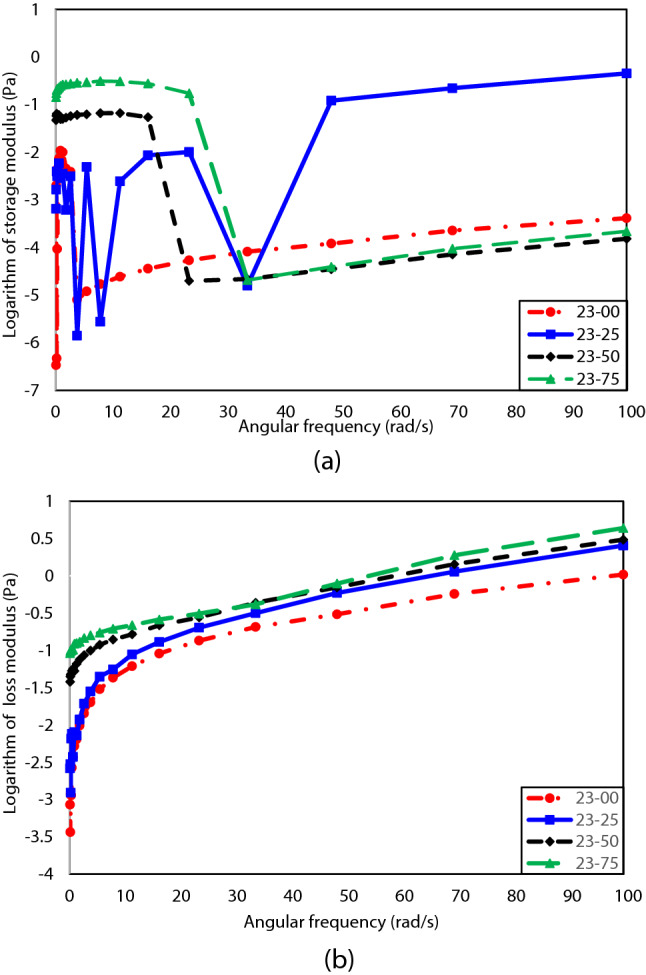
(**a**) Storage modulus vs. angular frequency for different concentrations of n-heptane in oil type 23. (**b**) Loss modulus vs. angular frequency for different concentrations of n-heptane in oil type 23.

The storage and loss modulus for oil type 23 in the presence of different concentrations of n-heptane are illustrated in Fig. 5(a) and (b), respectively. Although the solid-like behavior of oil type 23 (i.e., storage modulus) shows a complex trend by n-heptane concentration, it can be generally said that n-heptane increases the storage modulus and strengthen the elastic behavior at low angular frequencies. For experiencing a better operation condition, it is better to use a 25% volume concentration of n-heptane at the low angular frequency and 50% at the high angular frequency. In this condition, oil type 23 has the weakest solid-like behavior.

Figure 5(b) confirms that increasing the n-heptane concentration improves the samples’ loss modulus and liquid-like behavior for all angular frequencies. Indeed, it is better to add a 75% volume concentration of n-heptane to oil type 23 to experience the most substantial liquid-like behavior. Furthermore, increasing the angular frequency tends to strengthen the liquid-like manner and weaken the solid-like behavior of the considered oil sample. It was mentioned previously that the loss modulus describes the liquid-like behavior. Figure 5(b) shows that increasing the angular frequency continuously increases the loss modulus. The increase in the loss modulus is a good reason that justifies strengthening the samples’ liquid-like manner.

**Figure 6:**
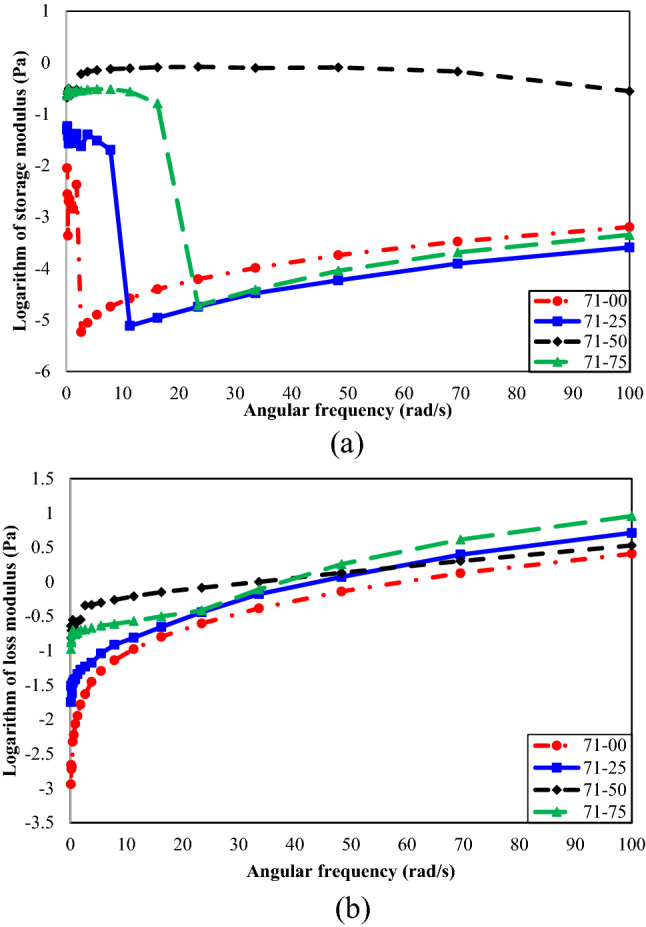
(**a**) Effect of n-heptane concentration on storage modulus vs. angular frequency of oil type 71. (**b**) Loss modulus vs. angular frequency for different concentrations of n-heptane in oil type 71.

Figure 6(a) and (b) presents the effect of n-heptane concentration on the storage and loss modulus of oil type 71, respectively. Figure 6(a) justifies that the angular frequency tends to weaken the solid-like behavior (storage modulus) of all samples containing different concentrations of n-heptane in oil type 71. The effect of n-heptane concentration on the solid-like behavior of oil type 71 is too complex to have a general trend. Generally, it seems that the samples 71–00 and 71–25 present the lowest storage modulus at low and high angular frequencies, respectively. In these situations, the oil samples show the weakest solid-like behavior.

It can be easily found from Fig. 6(b) that the angular frequency continuously strengthens the loss modulus and liquid-like behavior of oil type 71. Furthermore, a 50% volume concentration of n-heptane results in the strongest liquid-like behavior at the low angular frequency. In comparison, 75% is the best volume concentration for the high angular frequency. In this condition, oil type 71 has the highest value for the loss modulus, and its liquid-like behavior prevails.

Moreover, the angular frequency increases the viscous portion and decreases the elastic portion of the viscoelastic behavior. It can be said that the angular frequency strengthens the liquid-like behavior of oil type 71 more than its solid-like manner.

#### Investigation of the loss factor

This section analyzes the loss factor to monitor the effect of angular frequency and n-heptane concentration on the relative strength of liquid to solid-like behavior of the oil samples. As mentioned earlier, the loss factor shows the loss to the storage modulus ratio. A small loss factor indicates that the elastic portion controls a fluid viscoelastic behavior (solid-like behavior is more substantial than liquid-like). On the other hand, a high loss factor states that the viscous behavior controls the fluid’s viscoelastic behavior (liquid-like behavior prevails the solid-like one). By monitoring the loss factor, it is possible to find out whether any phase transitions happen in the system or not. A sudden change in the loss factor shows that the sample’s characteristics have changed during the test.

**Figure 7:**
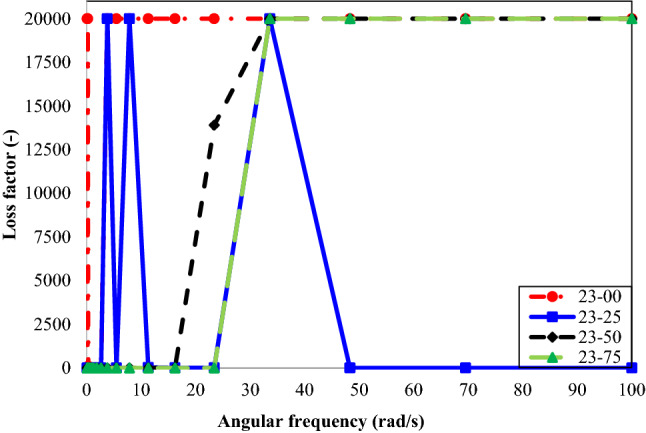
Loss factor vs. angular frequency for different concentrations of n-heptane in oil type 23.

**Figure 8:**
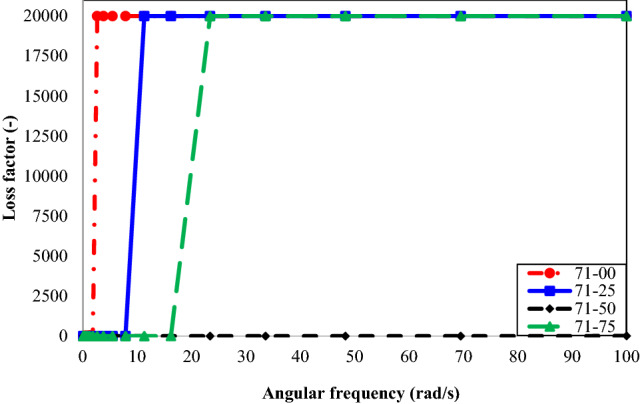
Loss factor vs. angular frequency for different concentrations of n-heptane in oil type 71.

The loss factor as a function of angular frequency for oil types 23 and 71 containing different volume concentrations of n-heptane is presented in Figs. 7 and 8, respectively. Increasing the angular frequency changed the samples’ viscoelastic behavior from solid-like to liquid-like.

Figure 7 shows that the oil sample of 23–25 has a reversed trend at an angular frequency higher than ~ 32 rad/s. Figure 5 showed that the storage modulus (G′) of the oil sample of 23–25 has suddenly increased at an angular frequency of higher than ~ 32 rad/s. It means that the asphaltene has re-dissolved in the oil and increased its solid-like behavior. It is also possible to consider this observation as an experimental or human error.

## Conclusion

Crude oil contamination by asphaltene increases the viscosity and transportation costs and leads to several problems in processing equipment. Therefore, this study focused on investigating the effect of n-heptane concentration and angular frequency on asphaltene precipitation and the viscoelastic behavior of oil samples from the Mansouri field, Iran. The viscosity test approved that all oil-heptane mixtures show the Newtonian behavior, and their viscosity remains constant at almost all shear rates. In addition, 75% is the best volume concentration of n-heptane to add the considered oil samples. This n-heptane concentration reduced the viscosity of oil types 23 and 71 by 97.2% and 96.3%, respectively. The frequency experiments approved that it is possible to improve the liquid-like behavior of all oil samples by applying the suitable angular frequency. An abnormal increase in the loss factor of oil sample 23 containing 25% volume percent of n-heptane at the angular frequency of higher than ~ 32 rad/s may be associated with the experimental error or human mistake. The frequency test showed that the angular frequency for oil types 23 and 71 should be higher than 33.6 and 23.4 rad/s, respectively. In these angular frequencies, the liquid-like behavior of the considered oil samples is more substantial than their solid-like manner.
